# Do Executive Functions Predict Binge-Drinking Patterns? Evidence from a Longitudinal Study in Young Adulthood

**DOI:** 10.3389/fpsyg.2017.00489

**Published:** 2017-03-31

**Authors:** Ragnhild Bø, Joël Billieux, Line C. Gjerde, Espen M. Eilertsen, Nils I. Landrø

**Affiliations:** ^1^Clinical Neuroscience Research Group, Department of Psychology, University of OsloOslo, Norway; ^2^Integrative Research Unit on Social and Individual Development, Institute for Health and Behavior, University of LuxembourgLuxembourg, Luxembourg; ^3^Laboratory for Experimental Psychopathology, Psychological Sciences Research Institute, Université catholique de LouvainLouvain-La-Neuve, Belgium; ^4^Department of Psychology, University of OsloOslo, Norway; ^5^Department of Mental Disorders, Norwegian Institute of Public HealthOslo, Norway

**Keywords:** binge drinking, executive functions, decision-making, young adults, longitudinal study

## Abstract

**Background:** Impairments in executive functions (EFs) are related to binge drinking in young adulthood, but research on how EFs influence future binge drinking is lacking. The aim of the current report is therefore to investigate the association between various EFs and later severity of, and change in, binge drinking over a prolonged period during young adulthood.

**Methods:** At baseline, 121 students reported on their alcohol habits (Alcohol use disorder identification test; Alcohol use questionnaire). Concurrently, EFs [working memory, reversal, set-shifting, response inhibition, response monitoring and decision-making (with ambiguity and implicit risk)] were assessed. Eighteen months later, information on alcohol habits for 103 of the participants were gathered. Data were analyzed by means of multilevel regression modeling.

**Results:** Future severity of binge drinking was uniquely predicted by performance on the Information sampling task, assessing risky decision-making (β = -1.86, 95% CI: -3.69, -0.04). None of the study variables predicted severity or change in binge drinking.

**Conclusion:** Future severity of binge drinking was associated with making risky decisions in the prospect for gain, suggesting reward hypersensitivity. Future studies should aim at clarifying whether there is a causal association between decision-making style and binge drinking. Performance on all executive tasks was unrelated to change in binge drinking patterns; however, the finding was limited by overall small changes, and needs to be confirmed with longer follow-up periods.

## Introduction

Binge drinking is a drinking pattern characterized by repeated episodes of intense alcohol consumption, leading to high levels of inebriation (Courtney and Polich, 2009). The drinking pattern may increase the risk of developing AUD (Olsson et al., 2016), a disorder which is developed in young adulthood by the majority of its sufferers (Kessler et al., 2005). This age period also coincides with the highest prevalence of binge drinking (Plant et al., 2009). Since AUD and binge drinking are associated with severe consequences (Rehm et al., 2010), it is important to identify potential risk factors that could be relevant when developing interventions targeting escalation of troublesome drinking patterns.

In several cross-sectional studies, reduced EFs are identified as risk factors for continued binge drinking among young adults (18–25 years of age) (Townshend and Duka, 2005; Goudriaan et al., 2007, 2011; Parada et al., 2012; Townshend et al., 2014; Bø et al., 2015, 2016; Banca et al., 2016). These studies indicate that young adult binge drinkers have aberrations in risky and ambiguous decision-making, working memory, inhibition, and response monitoring. Whether these aberrations are predispositions or consequences of alcohol use is not yet known. However, prospective studies in adolescent populations have identified aberrations in prefrontal functions, both as a predisposition for, and as a consequence of, initiating heavy alcohol consumption (Squeglia and Gray, 2016).

Executive performance is supported by the PFC (Miller and Cohen, 2001), an area particularly vulnerable to the neurotoxic effect of alcohol (Lyvers, 2000). In order to support self-control and goal-directed behaviors, the PFC orchestrates and maintains patterns of activity that represent goals and the means to achieve them. Many accounts describe what the underlying executive processes are. Some argue for a distinction between “cold” and “hot” EFs (Zelazo and Müller, 2002), referring to mechanistic and logically based processes, and processes requiring regulation of emotion, motivation, and reinforcement, respectively. While cold aspects are associated with the functioning of the dorsolateral PFC, hot aspects are associated with the functioning of the orbitofrontal cortex (Kerr and Zelazo, 2004).

Several attempts have been made to isolate the specific processes of cold prefrontal functions. Miyake et al. (2000) have, by means of a latent variable analysis of commonly used EF tasks, defined three separate, albeit correlated factors of cold EF: working memory (maintain/update), shifting, and inhibition. On the other hand, hot EF tasks trigger the need to monitor the self and the situation, and to regulate affect and motivation accordingly. These processes are, amongst others, captured by decision-making tasks (Damasio, 1994; Bechara et al., 1999), where immediate gains need to be set aside in order to achieve long-term goals. While dissociable, the cold components of EFs are still important to the hot processes (e.g., decision-making), and some errors in the hot EFs are partially traceable to the ineffectiveness of different cold control processes (Billieux et al., 2010; Del Missier et al., 2012).

In order to identify whether EFs are relevant predictors of future binge drinking, longitudinal studies are required. However, at present, studies in young adult populations are scarce. In a rather small sample of predominantly females, facets of inhibition predicted total number of intoxications and hangover days over a 28-day period, but not a composite binge score (Paz et al., 2016). In males, but not females, Goudriaan et al. (2011) found that binge drinking 2 years post testing was associated with disadvantageous, ambiguous decision-making. Aberration in this domain was also characteristic of the high severity binge drinking group at baseline compared to the low severity binge drinkers (Goudriaan et al., 2007). No association between binge drinking and response inhibition was detected for either gender at either time point. In a study investigating the role of intention to drink and EFs in young adult students, Mullan et al. (2011) found that planning and inhibition interacted with intention in predicting binge drinking the following week (defined by 5+ drinks per session). However, EFs (i.e., planning, decision-making, inhibition, set shifting) explained no significant variance. To date, these longitudinal studies are scarce and are mainly characterized by their coverage of only a few EF factors. Hence, at present, we are left with a fragmented picture of the exact relation between EFs and future binge drinking, and risk factors identified in cross-sectional studies (i.e., response monitoring, working memory, risky decision-making) are left unaccounted for as of now.

In contrast to the lack of longitudinal studies conducted in young adulthood, several prospective studies have addressed the relation between future alcohol use and EFs in adolescent populations. Crucially, these studies have shown abnormal brain activation during response inhibition as a consistent marker of transitioning toward alcohol abuse and binge drinking (Norman et al., 2011; Mahmood et al., 2013; Wetherill et al., 2013a,b; Whelan et al., 2014; Squeglia and Gray, 2016). With regard to neuropsychological vulnerabilities, preexisting deficits in working memory and inhibition have been found to predict increased alcohol use and first binge drinking episode in adolescence (Khurana et al., 2013; Peeters et al., 2015; Squeglia and Gray, 2016). Both adolescent groups who progressed into binge drinking and those who continued binge drinking have been reported as suffering from pre-existing poor decision-making skills (Xiao et al., 2009). In high-risk children, poor response inhibition—not set-shifting and working memory—has been emphasized as a risk factor for further problem drinking in adolescence (Nigg et al., 2006). Overall, it thus appears that vulnerabilities in facets of both cold and hot EFs constitute established risk factors for initiating and perpetuating (heavy) alcohol consumption and binge drinking during adolescence.

Though prospective existing studies suggested that performance on executive tasks are important risk factors for future binge drinking, these studies are not readily generalizable to young adult populations. Indeed, during the adolescent years, the prefrontal areas of the brain mature (Casey et al., 2000) and this development is associated with a decrease in risky behavior (Steinberg, 2004; Reyna and Farley, 2006). Therefore, EFs might be differently associated with future binge drinking in young adulthood compared to the association between EFs and the initiation of alcohol use during the adolescent years. Accordingly, onset and sustained binge drinking has been found to hold different risk factors (Copeland et al., 2012). Clearly, in order to improve the tailoring of prevention efforts in young adulthood, broader studies should be conducted in the young adult population.

Binge drinking is often operationalized in terms of consumption of a certain number of drinks within a limited time period (e.g., NIAAA, 2004), as a proxy for intoxication. However, it has been suggested that asking directly about subjective intoxication (i.e., drunkenness) might provide a better estimate of a heavy drinking episode (i.e., binge drinking), as it takes into account the level of tolerance (Andreasson, 2016) and other individual characteristics known to influence intoxication levels (e.g., metabolism, body composition, and gender). In order to overcome limitations associated with cut off (e.g., no valid cut off available); we decided to operationalize binge drinking as a continuous variable based on subjective drunkenness and speed of drinking.

To tackle the lack of longitudinal studies in the young adult population, we reassessed binge drinking in a sample of young adults 18 months after assessment of executive functioning. In alcohol studies, the EF tasks we employed are commonly used (Day et al., 2015). Thus, the main aim of the present study was to establish whether EFs are: (1) associated with future severity of binge drinking, and (2) associated with change in binge drinking patterns within young adulthood. Several hypotheses can be derived from the few available prospective studies. First, we expect working memory performance to be associated with future binge drinking. Second, based on longitudinal-, prospective-, and cross-sectional studies, we hypothesize that less advantageous and risky decisions will be related to future binge drinking. However, based on the inconsistent or null results obtained from previous studies, we do not expect inhibition and shifting abilities to predict future binge drinking. Association between change in binge drinking patterns and EFs has not previously been studied in a young adult population, and this research is therefore of an exploratory nature.

## Materials and Methods

### Participants and Procedure

One hundred twenty-one students (62 females) self-enrolled to a study of alcohol habits in a student population aged 18–25 (mean = 21.7, *SD* = 2.1). At baseline, they were all screened for serious physical and psychological health conditions, as described in Bø et al. (2015), and all reported regular alcohol consumption (AUDIT ≥ 1). They completed an online questionnaire about alcohol habits. Upon arrival at the Department of Psychology at the University of Oslo, all participants received both written and oral information about the project and their right to withdraw at any time. Informed consent was obtained by signature. Participants then underwent a short demographic interview and neuropsychological testing (T1). Upon testing, all self-reported abstinence from caffeine and nicotine for a minimum of 3 h, alcohol for 48 h, and all types of illegal substances for 7 days. At baseline, 119 participants agreed to participate in the follow-up study. Eighteen months later (T2), we contacted the participants by email and SMS, requesting them to complete an online questionnaire about their current drinking pattern. One hundred and three participants (50 females) completed the follow-up (85.1%). Data collection began in June 2013 and follow-up ended in February 2016. The study was conducted in compliance with the Helsinki Declaration and the Ethical principles for Nordic psychologists, as issued by the Norwegian Psychological Association. Upon completing the baseline assessments, participants obtained a gift card worth 250 NOK ($30). See **Table 1** for a description of the sample.

**Table 1:** Descriptives of the study sample.

	T1 Baseline			T2 18 months follow-up			
	*M*	*SD*	(Min–max)	*M*	*SD*	(Min–max)	*t*-statistics
Binge score	25.6	17.7	(1.32–99)	21.6	16.6	(1.32–88)	*t*(102) = 3.259, *p* = 0.001, *d* = 0.273
AUDIT	10.0	5.7	(1–27)	9.3	5.4	(0–29)	*t*(102) = 1.530, *p* = 0.129
Weekly alcohol consumption (units)	6.6	6.9	(0–32.5)	5.4	6.0	(0–25)	*t*(101) = 3.07, *p* = 0.003, *d* = 0.185

*M, mean; *SD*, standard deviation; AUDIT, alcohol use disorder identification test*.

### Alcohol Consumption

The last three questions of the AUQ [(10) Number of drinks per hour; (11) Number of times intoxicated by alcohol; (12) Percentage of time drunk when going out drinking] (Mehrabian and Russell, 1978) were used to calculate binge score (Townshend and Duka, 2002, 2005), which gives an estimate of binge drinking severity. The AUQ binge score is a validated (Townshend and Duka, 2002, 2005) and widely used method for exploring binge drinking (e.g., Kessler et al., 2013; Townshend et al., 2014; Czapla et al., 2015). As described previously (Bø et al., 2015, 2016), we employed a continuous approach to binge drinking, which is in line with the view of Enoch (2006). This operationalization is sensitive to an individual’s level of intoxication, and has the advantage of separating drinking pattern from overall alcohol consumption (Townshend and Duka, 2002). It is tangent to the NIAAA (2004, p. 3) view, where binge drinking is defined as “a pattern of drinking alcohol that brings BAC to 0.08 gram percent or above.” This level of intoxication is not always reached by a predefined number of drinks due to individual differences in metabolism, body composition, tolerance, and lack of specified duration of consumption (Thombs et al., 2003). Thus, self-reported drunkenness (i.e., loss of coordination, nausea and/or inability to speak clearly) overcomes the limitation associated with a predefined number of drinks.

The AUDIT (Saunders et al., 1993), a 10-item self-report questionnaire, was used to assess hazardous alcohol consumption during the last year. Participants also reported weekly alcohol consumption. These variables do not appear in the main analyses, as they do not directly assess binge drinking, but were included to present a detailed description of participants’ alcohol habits.

### Executive Functions

Working memory was assessed by the LNS task from the Wechsler Adult Intelligence Scale – Fourth edition (Wechsler, 2008). The participants were presented orally with a combination of letters and numbers. The task was to repeat the numbers in ascending order, followed by the letters in alphabetical order (e.g., 9-L-2-A; correct response is 2-9-A-L). The variable of interest was the maximum letter-number sequencing span.

Reversal and set-shifting were assessed by the IED from CANTAB^®^ (Cambridge Cognition, 2006). The task requires participants to learn, via computer assisted feedback, which of two presented stimuli is correct; pink shapes or white lines. After six consecutive, correct responses, the previously correct response is no longer rewarded, thus requiring the participants to switch from the old set to a new one. First, the change occurs intra-dimensionally (between pink shapes), then extra-dimensionally (between shapes and lines). The test terminates if the participant fails to reach the criterion of learning after 50 consecutive trails, or when the nine stages are completed. The variables of interest were the number of errors on trials before the extra-dimensional shift (reversal), and the number of errors on trials after the extra-dimensional shift (set-shift).

Decision-making under explicitly presented risk was assessed by the IST from CANTAB^®^ (Cambridge Cognition, 2006). In a series of 10 trials, the participants were required to consecutively open boxes in a 5 × 5 matrix that revealed colored squares, and then subsequently decide which of the two colors lay in the majority. The color of the boxes was changed in every trial. A conflict between reinforcement and certainty was present as the possible gain of 250 points was reduced by 10 for every box opened. To maximize reinforcement, the test taker must tolerate a high degree of uncertainty, because sampling information until a point of high certainty would yield very few points. In case the wrong color was chosen, 100 points were lost irrespective of number of boxes opened. The variable of interest was the mean probability of being correct at the time of decision (see Clark et al., 2006 for a comprehensive description of the computed index).

Pre-potent response inhibition and response monitoring were both assessed by the SST from CANTAB^®^ (Cambridge Cognition, 2006). A practice block of 16 go-trials (right or left facing arrow requiring corresponding response on a press pad) preceded the main task, which consisted of 320 trials. In a minority of these (∼25%), an auditory beep (the stop signal) indicated that the response should be withheld on that particular trial, thereby assessing the ability to inhibit an already initiated motor response (Logan, 1994). The delay ahead of the stop signal (stop signal delay; SSD) was adjusted according to performance. Over time, this tracking procedure stabilized the probability of successful inhibition around 0.5 for each participant. We quantified the pre-potent response inhibition process by computing the SSRT using the so-called ‘integration approach.’ This method aims to minimize false skewing of the SSRT that may result from continuous slowing on go-trials (Verbruggen et al., 2013). In this approach, reaction times on go-trials are rank-ordered individually for each participant in each of the five blocks. Then we subtracted the mean SSD from the *n*th percentile of the reaction time on go-trials corresponding to the percentage of unsuccessful stop-trials in the particular block, yielding the SSRT for this block. The mean SSRT across all five blocks was the variable of interest. Response monitoring, referring to the ability to evaluate action outcomes and let feedback guide future performance (Thakkar et al., 2014), was investigated by means of PES. PES was calculated by contrasting reaction times for “Go- after-go” trials and “Go-after-failure to stop” trials, as described in Lawrence et al. (2009).

Decision-making under ambiguity and implicitly presented risk was estimated by the computerized version of the IGT (Bechara et al., 1999). The participants were required to draw cards from one out of four decks of cards (A, B, C, and D), and the task instruction was to maximize profit. Unbeknownst to the participant, two of the decks (C and D) resulted in overall gain, whereas the others resulted in overall loss. The task consisted of five blocks of twenty trials. The last forty trials (trials 61–100) were proposed to measure decision-making under implicitly presented risk (because the reinforcement contingences were at least partly known), and the first forty trials (trials 1–40) dealt with decision-making under ambiguity (Brand et al., 2006; Billieux et al., 2010). The variable of interest was the number of advantageous decisions (decks C+D)–(decks A+B) in trials 1–40 (decision under ambiguity) and trials 61–100 (decision under implicitly known risk), respectively.

Please see **Table 2** for overview of study variables. All computerized tests were administrated on a Dell Latitude D610 laptop computer with a 14.1′′ LCD screen using 1024 pixels × 768 pixels at 32-bit color quality. Press pad, touch screen, and external speakers were connected. An internal mouse pad was used to obtain responses on the IGT. The EF-tests were administrated in a pre-determined fixed order (corresponding to the order in which the tasks are described in the section “Materials and Methods,” see above).

**Table 2:** Overview of study variables.

Task	Variable	Construct measured
Letter number sequencing task	Letter number sequencing span	Working memory; maintain and update
Intra-extra dimensional shift	Errors before extra-dimensional shift	Reversal
	Errors after extra-dimensional shift	Set-shifting
Stop signal task	Stop signal reaction time	Prepotent response inhibition
	Post error slowing	Response monitoring
Information sampling task	*p*(correct)	Decision-making under explicitly presented risk
Iowa gambling task	Advantageous choices trials 1–40	Decision-making under ambiguity
	Advantageous choices trials 61–100	Decision-making under implicitly presented risk

### Statistical Analysis

All statistical analyses were performed with IBM SPSS 22 and Stata 14. Due to technical problems, CANTAB^®^-data for three participants were missing. One male participant, who had previous detailed knowledge about the test, did not perform the IGT. Five participants completed all cards in one deck (60 cards) during the fourth block of the IGT, forcing an unintended change in strategy. The IGT data from these participants were therefore discarded from analysis. Binge scores were logarithmically transformed due to skewed distributions.

Independent sample *t*-tests were conducted for all study variables to detect significant group differences between participants taking part at both time points of the study and those participating only at baseline. Pairwise comparisons between alcohol consumption measures at T1 and T2 was calculated. Bivariate correlations were computed to investigate the relation between T1 binge drinking and executive performance, and the relation among predictor variables. Partial correlations between T2 binge drinking and executive performance, controlling for T1 binge drinking, were calculated. Due to the exploratory nature of the present study, corrections for multiple comparisons were not employed. Employing a more stringent criterion for alpha would increase the risk for committing type II errors. Because the aim of the study is to identify risk factors, the cost associated with overlooking potentially important risk factors could be substantial.

**FIGURE 1:**
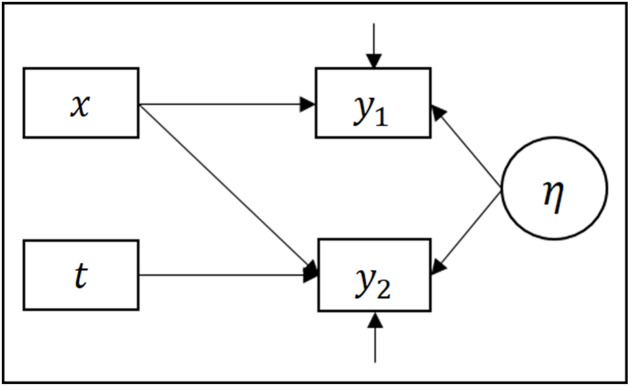
**Path diagram illustrating key components of the statistical models**. Observed variables are represented by rectangles and latent variables by ovals. y_1_ and y_2_ represents binge-severity at time-point 1 and 2, respectively. *x* represents all explanatory variables except time, which is represented by t. The arrows represent regression effects.

Due to the longitudinal data collection, we used a linear multilevel model with a random intercept over participants to allow for dependence in responses within participants. Self-reports of alcohol consumption across time are correlated, and treating them as independent observations could lead to incorrect estimates of standard errors. This model allows for inclusion of participants with missing responses at the second occasion. **Figure 1** illustrates key components of the statistical model. Parameters were estimated according to the maximum likelihood criterion. Our analytical approach proceeded in three steps. First, we estimated a null model without any of the covariates of interest; second, we included main effects of all covariates; finally, we also included interactions allowing all covariate effects to vary between baseline and follow-up. In order to investigate our first aim, that is whether any of the EFs were related to severity in binge drinking at the follow-up, we compared the first and the second model by means of a likelihood ratio test. Using this test, the null hypothesis that all covariate effects were equal to zero was evaluated. To investigate our second aim, which was to test whether any of the EFs were related to change in the binge severity between baseline and follow-up, we compared the second and the third model, testing the null hypothesis that all interaction effects were equal to zero.

## Results

In **Table 1**, socio-demographic characteristics and alcohol consumption habits of the study sample are reported. A significant decline in binge score and weekly alcohol consumption was detected.

The two participants who refused to be contacted at follow-up differed from those agreeing to be contacted: gender (equal variances not assumed): *t*(118) = 11.329, *p* < 0.001; IED errors after extra-dimensional shift: *t*(117) = -2.509, *p* = 0.013; IGT advantageous choices trials 61–100: *t*(113) = 2.324, *p* = 0.022; IST *p*(correct): *t*(115) = 2.111, *p* = 0.037. Overall, however, participants attending follow-up did not differ from those who only participated at baseline on any demographic [age: *t*(119) = -1.131, *p* = 0.260; gender: *t*(119) = 1.419, *p* = 0.158], drinking [binge score: *t*(119) = 0.107, *p* = 0.915; AUDIT: *t*(119) = 0.045, *p* = 0.065; weekly alcohol consumption: *t*(119) = -0.888, *p* = 0.376], or neuropsychological variables [LNS: *t*(119) = 0.418, *p* = 0.677; IED errors before extra-dimensional shift: *t*(117) = 0.439, *p* = 0.66; IED errors after extra-dimensional shift: *t*(117) = 0.219, *p* = 0.827, SSRT: *t*(117) = -0.577, *p* = 0.565; PES: *t*(117) = -0.164, *p* = 0.870; IGT advantageous choices trials 1–40: *t*(117) = -0.715, *p* = 0.476, IGT advantageous choices trials 61–100: *t*(113) = -0.1.202, *p* = 0.232; IST *p*(correct): *t*(115) = -1.540, *p* = 0.126]. Accordingly, the dropout was non-systematic.

Bivariate and partial correlations between the predictors and the binge scores at baseline and follow-up are presented in **Table 3**.

**Table 3:** Correlations between binge scores and executive functions.

	T1 binge score	T2 binge score
Letter number sequencing span	0.115	0.127
SST stop signal reaction time	-0.029	-.016
SST post error slowing	-0.184ˆ*	0.081
IED errors before extra-dimensional shift	-0.036	0.066
IED errors after extra-dimensional shift	0.060	0.062
IST *p*(correct)	-0.196ˆ*	-0.111
IGT advantageous choices trials 1–40	0.129	0.105
IGT advantageous choices trials 61–100	-0.044	0.021

^∗^*p < 0.05. IED, intra extra dimensional shift; IGT, Iowa gambling task; IST, information sampling task; SST, stop signal task. Partial correlations between binge score at T2 and executive functions, controlling for T1 binge score*.

Bivariate correlation between the measures of EFs are presented in **Table 4**.

**Table 4:** Bivariate correlations between behavioral measures.

	2	3	4	5	6	7	8
(1) Letter number sequencing task	-0.118	-0.110	-0.122	-0.039	0.055	0.138	0.098
(2) IED errors before extra-dimensional shift		0.000	0.029	0.129	0.046	-0.239^∗^	-0.026
(3) IED errors after extra-dimensional shift			-0.026	-0.101	-0.074	-0.172	-0.013
(4) SST stop signal reaction time				0.155	-0.023	-0.002	-0.001
(5) SST post error slowing					0.003	0.077	-0.005
(6) IGT trials 1–40						0.309^∗∗^	0.054
(7) IGT trials 61–100							0.201^∗^
(8) IST *p*(correct)							

^∗^*p < 0.05, ^∗∗^*p* < 0.001. IED, intra extra dimensional shift; IGT, Iowa gambling task; IST, information sampling task; SST, stop signal task*.

The null model, including only a constant term for the fixed effects, showed substantial correlation (intraclass correlation = 0.63, 95% CI = 0.51, 0.74) in the responses within participants. The likelihood ratio test, comparing the null model with the second model, showed significant improvement in fit after inclusion of all covariates [χ^2^ (10) = 35.00, *p* < 0.01]. Comparing the second and third model, there were no improvements in fit by inclusion of any interaction terms [χ^2^ (8) = 5.27, *p* = 0.73]. We therefore proceeded by interpreting the coefficients from the second model. There was significantly higher mean scores at baseline than follow-up (β = 0.22, 95% CI = 0.09, 0.34). Further, females on average scored lower than males (β = -0.41, 95% CI = -0.68, -0.14). Risky decision-making under explicitly presented risk (IST) was negatively related to binge drinking severity (β = -1.61, 95% CI = -3.19, -0.03). None of the other variables of interest were significantly related to binge drinking severity. Please see **Table 5** for a detailed account of the estimated model.

**Table 5:** Results from the multilevel modeling.

Model	Model 1: Main effects		Model 2: Change	
Fixed effects	β (SE)	95% CI	β (SE)	95 % CI
*Intercept*	*3.68 (0.75)*	*2.22, 5.14*	*3.40 (0.87)*	*1.70, 5.10*
Sex	-0.41 (0.14)	-0.67, -0.14	-0.41 (0.14)	-0.68, -0.15
Time	0.22 (0.07)	0.09, 0.34	0.72 (0.77)	-0.80, 2.23
IED errors before extra-dimensional shift	0.01 (0.01)	-0.00, 0.02	0.01 (0.01)	-0.00, 0.03
IED errors after extra-dimensional shift	-0.01 (0.01)	-0.00, 0.02	-0.00 (0.03)	-0.06, 0.06
IST *p*(correct)	-1.61 (0.81)	-3.19, -0.03	-1.86 (0.93)	-3.69, -0.04
Letter number sequencing span	0.08 (0.06)	-0.04, 0.21	0.12 (0.07)	-0.03, 0.26
SST stop signal reaction time	-0.00 (0.00)	-0.00, 0.00	-0.00 (0.00)	-0.00, 0.00
SST post error slowing	-0.00 (0.00)	-0.00, 0.00	-0.00 (0.00)	-0.00, 0.00
IGT advantageous choices 1–40	0.02 (0.01)	-0.01, 0.04	0.02 (0.01)	-0.00, 0.05
IGT advantageous choices 61–100	-0.00 (0.01)	-0.01, 0.01	-0.00 (0.01)	-0.02, 0.01
				
Time × IED errors before extra-dimensional shift	-	-	-0.01 (0.01)	-0.02, 0.01
Time × IED errors after extra-dimensional shift	-	-	-0.02 (0.03)	-0.07, 0.03
Time × IST *p*(correct)	-	-	0.47 (0.82)	-1.14, 2.08
Time × Letter number sequencing span	-	-	-0.06 (0.07)	-0.19, 0.07
Time × SST stop signal reaction time	-	-	-0.00 (0.00)	-0.00, 0.00
Time × SST post error slowing	-	-	-0.00 (0.00)	-0.01, 0.00
Time × IGT advantageous choices trials 1–40	-	-	-0.01 (0.01)	-0.03, 0.01
Time × IGT advantageous choices trials 61–100	-	-	-0.00 (0.01)	-0.02, 0.01
				
**Variance components**				
Intercept	0.31 (0.06)	0.21, 0.45	0.32 (0.06)	0.22, 0.46
Residual	0.22 (0.03)	0.16–0.29	0.20 (0.03)	0.15, 0.27

	**Log likelihood**		**Log likelihood**	

	-209.32		-206.69	

*IED, intra extra dimensional shift; IGT, Iowa gambling task; IST, information sampling task; SST, stop signal task*.

## Discussion

The current study investigated the association between EFs and future severity of and change in binge drinking among young adults over a period of 18 months. Results revealed that only decision-making under explicitly presented risks (IST) was associated with *future severity*. Since binge drinking is associated with potentially serious consequences, it is important to identify risk factors that can later be tested for causality in appropriate designs. No other measures of EFs were significantly associated with future severity. The latter result was unexpected, and suggests that findings obtained in adolescent samples are not readily generalizable to adult populations. This might be due to developmental factors affecting the occurrence of risky behavior in various age groups. Alternatively, the lack of significant associations might be due to different factors contributing to initiation vs. sustainment of binge drinking. Of note, some EFs, which have been established as impaired in previous cross-sectional studies on binge drinking, failed to predict future binge drinking in the current study. Although replications are required, our study thus contributed to detecting which specific EFs are the best candidates for specific preventions and early interventions. None of the variables included in this study were associated with *change* in binge drinking over an 18-month period, though this might have been due to the small changes in binge drinking during the period.

In this study, binge drinking was defined by the AUQ binge score; a continuous variable based on self-reported drunkenness and consumption speed. Accordingly, this definition might be better at capturing those at risk of alcohol related harm due to high BACs, compared to more traditional definitions based on number of drinks per occasion. We did not make any cut-off with regard to possible AUD. At follow-up, five participants had AUDIT scores ≥ 20, which is indicative of alcohol dependence (Babor et al., 2001). This proportion is probably quite representative of community samples where the 12-month prevalence rate of severe AUD in the age group 18–29 is 7.1% (Grant et al., 2015). Generally, the current sample consists of healthy, well-functioning, highly educated young adults, and it is not certain that the results will generalize to other samples. Therefore, the study should be replicated in broader populations to ascertain the generalizability of these current results.

### Future Severity of Binge Drinking

One of the variables associated with severity of binge drinking at baseline (e.g., Bø et al., 2016), was also associated with future severity of binge drinking. Specifically, decision-making under explicitly presented risks (IST) was associated with future severity of binge drinking, suggesting that more severe, future binge drinkers are driven by prospect for gain when making decisions. Accordingly, alcohol expectancies are shown to mediate frequency in alcohol consumption among college students, with those having the highest expectancies consuming the most (Brown et al., 1985). A decisional balance characterized by hyperactivation to reward and hypoactivation to punishment have previously been identified among persons with AUD (Shiv et al., 2005). According to the continuum hypothesis (e.g., Enoch, 2006; Lannoy et al., 2014), this type of decisional (im)balance might represent one of the relevant tracks for developing more serious alcohol use among binge drinkers.

In contrast to prior studies, ambiguous decision-making (IGT) was unrelated to future binge drinking. This might be due to the definition of binge drinking employed by Goudriaan et al. (2011), whose definition of binge drinking actually corresponds to heavy consumption rather than the drinking pattern. In addition, since the number of trials differed, and their finding was applicable to men only, strict comparisons across studies are not warranted. The construction of the IGT led to the exclusion of data from five participants. This is a known phenomenon (e.g., Goudriaan et al., 2007), and we have no reason to believe that this has affected the results with regard to ambiguous decision-making.

All cold EF variables were unrelated to future binge drinking. In accordance with previous findings (Goudriaan et al., 2011; Paz et al., 2016), response inhibition (SSRT) did not predict future binge drinking. Moreover, set-shifting (IED), response monitoring (PES), and working memory performance (LNS) were not associated with future binge drinking. These null-findings represents an important addition to the previously inexistent literature. The fact that cold EFs failed to predict future binge drinking in young adulthood contradicts previous findings obtained in adolescent populations. Improvements in reflective functions, associated with prefrontal maturation taking place in the period from adolescence to young adulthood, might be the reason for this difference. However, it is worth mentioning that while previous studies showed that cold EF deficits predict future heavy alcohol consumption and alcohol related problems in adolescence, these have most of the time been identified at the cerebral and not the behavioral level. Thus, future studies combining the use of neuroscience and behavioral measures are required to clarify the relation between EFs and future binge drinking.

Binge drinking has previously been conceptualized in a dual-process framework (Lannoy et al., 2014), suggesting that the behavior might be a product of an imbalance between affective-automatic and reflective processes. The fact that cold EFs failed to predict future binge drinking might imply that increased affective-automatic processes, rather than defective reflective processes, was contributing to an increased risk of engaging in binge drinking in the future. Thus, to understand how the decisional balance tips in favor of immediate gratification and risky behavior, future studies should aim at elucidating the exact nature of reward and punishment (hyper)sensitivity to the development in drinking pattern.

### Change in Binge Drinking

Neuropsychological function was not related to change in binge drinking habits in this sample, which could be considered positive, considering the documented negative effect binge drinking has on prefrontal neural functioning (Maurage et al., 2012). At an aggregate level, a significant decline in binge drinking over 18 months was detected. With age, binge drinking frequency is expected to decline (Skretting et al., 2015); however, the effect size was small. Perhaps the ability to change drinking pattern is more heavily reliant on the capacity in EFs when larger changes are required, e.g., due to increased social obligations and responsibilities when ending college. Studies with longer duration of follow-up are needed to clarify this. Moreover, we cannot rule out that the very act of taking part in the study led to the detected reduction in binge drinking.

In the current study, the measure of binge drinking behaviors relied on subjective accounts of drunkenness. However, subjective assessments of drunkenness are known to be potentially inconsistent over time (Kerr et al., 2006). This could perhaps—at least partly—explain the apparent reduction in binge drinking observed at T2 in our study. However, it is unlikely that an important change in definition actually occurred over the two periods of the study, especially because the AUQ provides examples of what drunkenness implies in this context.

Previous research has indeed identified different trajectories for binge drinking in the age period 18–24 (Schulenberg et al., 1996). Nearly 60% of the total sample of 9,945 participants continued binge drinking at the same levels, while trajectories in over 30% of the sample reflected discontinuity. Future studies should acknowledge this variation when investigating changes in drinking pattern, and should ideally include multiple time-points to account for random changes attributable to the selected period.

### Limitations

Some limitations should be acknowledged. First, we had a modest sample size, which gives limited statistical power to detect associations. Second, we did not have data on potentially important confounding variables (e.g., genetics, environmental), which may be relevant to drinking pattern development. Third, while the drinking culture in Norway is characterized by lower alcohol consumption compared to other European countries, the drinking pattern is rather hazardous (Rehm et al., 2010). Because drinking to intoxication is quite common, it might not be subject to social sanctions as it would in other cultures. Moreover, perception of drunkenness varies across countries (Muller and Schumann, 2011). In combination with the strict alcohol legislation, generalizations to other countries must be preceded by caution. Fourth, validity and reliability of self-reported alcohol consumption has been found to be at reasonable levels (Del Boca and Darkes, 2003); however, when comparing components of the binge score to diary accounts, the number of times drunk and number of drinks per hour were significantly under- and overestimated, respectively (Townshend and Duka, 2002). Thus, using other measures, like the timeline follow back in combination with AUQ binge score, might be more closely related to real-life consumption (Lake et al., 2015).

## Conclusion

The current study simultaneously investigated different factors of EFs in future severity and change in binge drinking in young adulthood. While future severity was predicted by decision-making focusing on the prospect for gain, none of the study variables was predictive of change in binge drinking, which could be related to the overall small aggregate change in this allocated period. In order to build preventive efforts aimed at reducing binge drinking, future studies should aim at investigating whether risky decision-making and binge drinking is causally related.

## Author Contributions

RB, JB, and NL designed the study. RB, LG, and EE analyzed the data. All authors contributed to the interpretation of the data. RB wrote the first draft, and all authors have revised it critically for intellectual content. The final version is approved for publication by all authors.

## Conflict of Interest Statement

The authors declare that the research was conducted in the absence of any commercial or financial relationships that could be construed as a potential conflict of interest.

## Abbreviations

AUD: alcohol use disorder
AUDIT: alcohol use disorder identification test
AUQ: alcohol use questionnaire
BAC: blood alcohol concentration
EFs: executive functions
IGT: Iowa gambling task
IST: information sampling task
LNS: letter number sequencing
PES: post error slowing
PFC: prefrontal cortex
SSD: stop signal delay
SSRT: stop signal reaction time
SST: stop signal task.
